# Pesticides and the evolution of the genetic structure of *Anopheles coluzzii* populations in some localities in Benin (West Africa)

**DOI:** 10.1186/s12936-019-3036-z

**Published:** 2019-12-05

**Authors:** Arsène Jacques Y. H. Fassinou, Come Z. Koukpo, Razaki A. Ossè, Fiacre R. Agossa, Roseric Azondékon, André Sominahouin, Casimir Kpanou, Hermann Sagbohan, Boulais Yovogan, Martin C. Akogbéto, Michel Sezonlin

**Affiliations:** 1 Centre de Recherche Entomologique de Cotonou (CREC), Cotonou, Benin; 2 Laboratoire Evolution Biodiversité des arthropodes et Assainissement, Faculté des Sciences et Techniques/Université d’Abomey-Calavi (FAST/UAC), Abomey-Calavi, Benin; 3 Ecole Doctorale Sciences de la Vie et de la Terre (EDSVT/FAST/UAC), Abomey-Calavi, Benin; 4 Université Nationale d’Agriculture (UNA), Porto-Novo, Benin

**Keywords:** *Anopheles coluzzii*, Genetic structure, Pesticide, Malaria, Resistance, Benin

## Abstract

**Background:**

Changes in the natural habitats of insect groups are determined the genetic polymorphisms between individuals. The objective of this study was to establish the genetic structure of the *Anopheles coluzzii* populations in four localities of Benin.

**Methods:**

Insecticide surveys and larval sampling were conducted on 4 study localities, including Cotonou, Ketou, Zagnanado, and Sô-Ava. Molecular characterizations were performed on the *Anopheles* mosquitoes collected with the allelic and genotypic frequencies of *kdr* gene determined. The multiple comparison Chi square test for proportions was performed with R version 3.3.3. Next, the observed heterozygosity, expected heterozygosity, and indices of fixation, and genetic differentiation were estimated. Finally, the Hardy–Weinberg equilibrium (EHW) was determined to assess whether panmixia exists in the different populations of mosquitoes of the agroecological zones under study.

**Results:**

Carbamates, pyrethroids, organophosphorus and organochlorines use have been reported in all localities except Sô-Ava. *Anopheles coluzzii* was strongly represented across all study localities. The *L1014F* allele was observed in the localities of Kétou, Cotonou and Zagnanado. Likewise, insecticide selection pressure of homozygous resistant individuals (*L1014F/L1014F*) was significantly higher in Kétou, Cotonou and Zagnanado (*p* value < 0.05). Surprisingly in Sô-Ava, a relatively high frequency of the *L1014F* allele despite the reported absence of pesticide use was observed. All mosquito populations were found to be deficient in heterozygosity across the study sites (*F*_*IS*_< *0*). No genetic differentiation (*F*_*ST*_< *0*) was observed in the localities of Zagnanado and Kétou.

**Conclusion:**

The survey on the use of insecticides showed that insecticide selection pressures differ across the investigated localities. It would be desirable to rotate or apply formulations of combined products with different modes of action. Doing so would enable a better management of resistant homozygous individuals, and mitigate the resistance effect of commonly used insecticides.

## Background

Vector-borne diseases are among the main causes of morbidity and mortality in humans and animals [1]. Out of the many vector-borne human pathologies, malaria stands out as it remains a major public health problem worldwide, with over 219 million cases estimated in 2017 [2]. In 2017, 15 of the 91 countries that reported cases of malaria, have also reported 80% malaria transmission rate. Unfortunately, these nations are mostly in sub-Saharan Africa, with the exception of India [2].

In the fight against malaria, two prevention methods are used: long-lasting insecticidal nets (LLINs) and indoor residual spraying (IRS) [3]. On the African continent, the use of pyrethroid-based insecticides in malaria control has increased considerably in recent years after series of LLINs and indoor spraying campaigns [4]. Materials impregnated with insecticide have proven relative effectiveness in almost all epidemiological profiles [5, 6].

In developing countries, urbanization and agriculture are factors that can contribute to population growth and, consequently, to the increase of malaria transmission rates [7]. Urbanization can increase malaria transmission following a poor implementation of the sanitation plan. For example, a lack of adequate water drainage system and an excessive distortion of the natural terrain due to human dwellings and community infrastructure may promote the emergence of mosquito breeding sites that are suitable environments for the reproduction of malaria vectors, the emergence of adult mosquitoes and subsequently promote human-vector contact [8]. Based on the soils, agricultural fields are also very good breeding sites for malaria vectors as they contribute to the selection of resistant individuals due to the uncontrolled use of multiple pesticides [9]. Malaria vector insects are able to develop multiple mechanisms of resistance to insecticides depending on their exposure to agricultural pollutants [10]. In the Republic of Benin, several studies have confirmed the role of pesticides in the development of insecticide resistance in *An. gambiae* [11, 12]. Insecticide resistance is, therefore, not a new phenomenon in this region. In 1999, it was reported that the main malaria vector, *An. gambiae* sensu stricto (*s.s.*) had already developed resistance to synthetic pyrethroids in Benin [13]. The massive use of insecticides in agriculture [14] and in public health [15, 16] is the main driver of the development of insecticide resistance in malaria vectors [17]. To this day, two main mechanisms are known to be involved in metabolic resistance to insecticides in mosquitoes. The mutations on the voltage-gated sodium channel gene that are the targets of pyrethroids, and the overproduction of detoxifying enzymes are the main mechanisms of mosquito resistance to insecticide [18–20].

Dispersion of point mutations in *Vgsc* gene in anopheline populations and other taxa that are phylogenetically close has been associated with the use of pyrethroids, which are the most commonly used against malaria vectors in the world [21].In sub-Saharan Africa, this mutation affects the *kdr* gene site *L1014* leading to a synonymous substitution of leucine to phenylalanine (*L1014F*) [21] on one side or leucine to serine (*L1014S*) [17] on the other.

Genetic polymorphism, a characteristic of living organisms, and gene flow between individuals of different populations can be caused in observed changes in the natural habitats of insect groups [22]. According to those studies, spatial variations may be linked, for example, to the splitting of an insular environment [22], to the particularities of some biotopes, such as cellars, ponds [23], or to strict infeodation to certain hosts [24]. Temporal variations in the availability of habitat resources, on the other hand, may affect the structure of the populations by influencing seasonal changes in numbers that occur through migration [25]. Ecology and demography are not to be ignored as they are key factors in the process of species distribution. Knowledge of the genetic structure of disease vector populations is essential to understanding vector capacity through the identification of the underlying factors and, by extension, to better assess the effectiveness of vector control programmes, as well as the implementation of new control strategies when needed [26, 27].

The main goal of this study was to establish the genetic structure of the populations of *An. coluzzii* collected from different agro-ecological zones in Benin. Specifically, to (i) gather an inventory of the different pesticides used in those zones; (ii) establish the genetic structure of *An. coluzzii* populations, and (iii) study the genetic and genotypic differentiations.

## Methods

### Study area

This study was conducted in the Republic of Benin, a West African country, located in the intertropical zone between the equator and the tropic of cancer, specifically the 6°30′ and 12°30′ parallels of North latitude on one hand and, and the meridians 1° and 3°40′ of eastern longitude, one the other hand. Based on pedoclimate factors and the diversity of crops grown in some localities, Benin is divided into eight agro-ecological zones [28]: the extreme north Benin, the Northern cotton zone, the South Borgou Food Zone, the Western Atacora Zone, the Center Cotton Zone, the Bar Land Zone, the Fishing Zone, and the Depression zone.

**Fig. 1:**
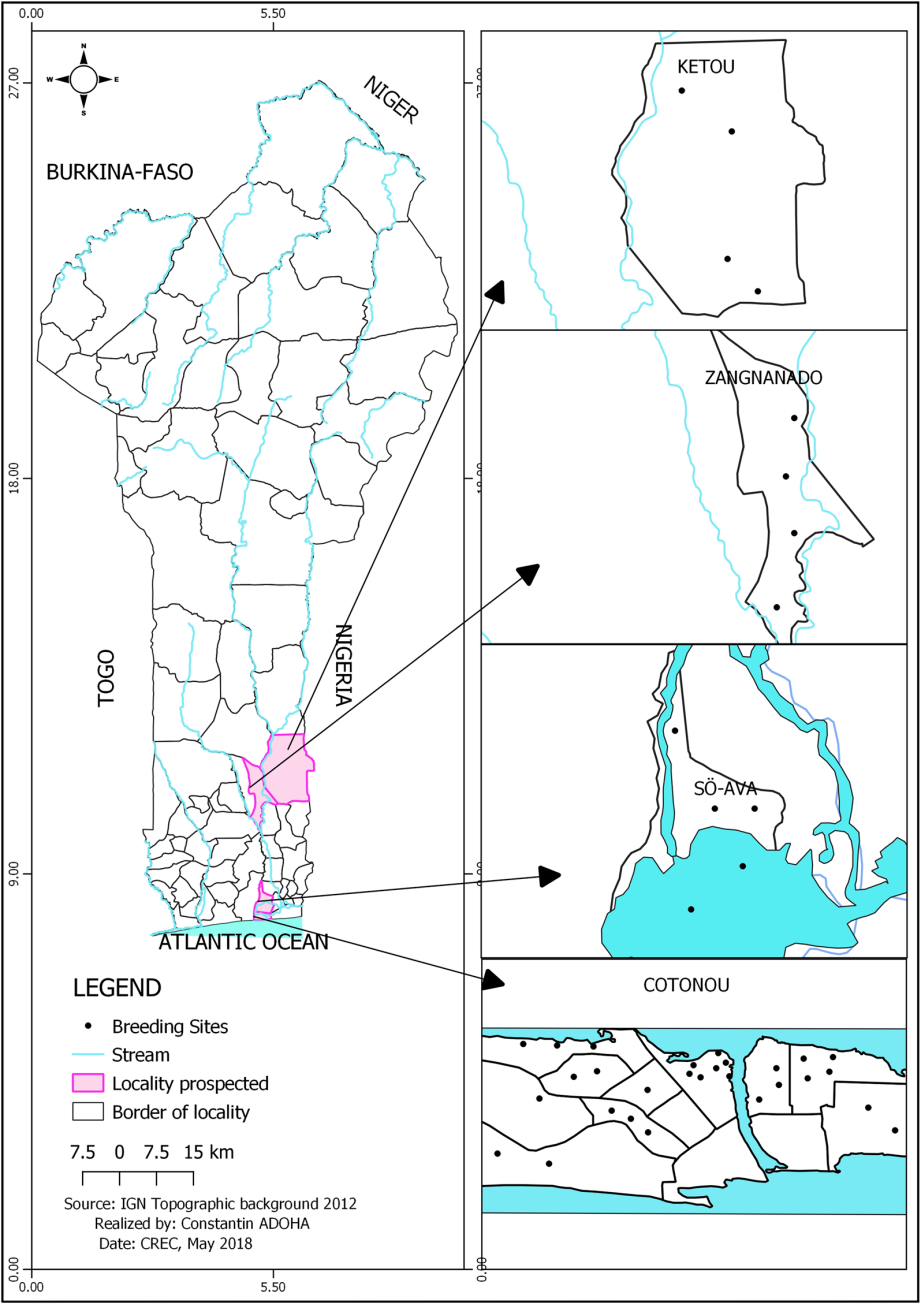
Map showing the different study localities

Mosquitoes used as biological material in this study were collected from the localities of Kétou (Cotton Zone of Center), Zagnanado (Bar Land Zone), Cotonou and Sô-Ava in the Fishing Zone. Figure 1 shows the study locations represented by many mosquito collections sites.

All prospected collection sites are located in southern Benin regardless of the zone. A diversity of agricultural practices is observed in the municipalities of Cotonou, Kétou and Zagnanado. In these localities, producers use pesticides to maximize their agricultural yield. Since the community of Sô-Ava is largely a lake region, the main activity practiced by residents is fishing in various forms with various gear. A survey of agricultural practices in the study localities identified the different families of insecticide used.

### Insecticide use survey

Surveys of the use of different insecticides used by farmers were carried out in the four study localities (Cotonou, Sô-Ava, Kétou, Zagnanado). The numbers of farmers available in each locality was determined using the second-generation communal development plan and the village notebooks of the departments of each locality. The number of farmers in Cotonou, Sô-Ava, Zagnanado and Kétou are approximately 10,864, 52,279, 34303 and 103,694, respectively [29–32]. The farmers were chosen according to a reasoned choice technique: in the different farming populations, children and women were not questioned because they are usually responsible for subsistence farming which does not require the use of pesticides. The remaining group consisted of large producers of cotton, cereals, tubers and market gardening products. The various crops grown and the number of farmers, survey respondents by municipality are recorded in Table 1.

**Table 1:** Distribution of survey respondents by municipality

Municipality	Type of farmers				
	Cotton producers	Vegetable producers	Tubercule producers	Cereal producers	Total
Cotonou		49	3	8	60
Kétou	34	5	6	15	60
Zagnanado	25	5	6	24	60
Sô-Ava		47	10	13	60
Total	59	106	25	50	240

Farmers have been found to grow a variety of products whose dominance varied from a municipality to another (Table 1). The frequency of use and the pesticide types used to improve yield depended on the culture and financial means available to each farmer. These factors limited the scope of this study as it was difficult to estimate the frequency of pesticide use.

### Mosquito sample collection

Anopheles larvae were collected from May 2015 to October 2017 during dry and rainy seasons in the localities initially selected using the “dipping” technique [33]. It consists of taking the mosquito larvae from the surface of the breeding site using a ladle. The harvested larvae of *An. gambiae* sensu lato (*s.l.*) were filtered and poured into different containers before being transported to the Insectarium of the “Centre de Recherche Entomologique de Cotonou” (CREC) for rearing. As reported in the entomological literature [34, 35], between 80 and 300 eggs are laid by the female *Anopheles* on the surface of the water. After the aquatic life cycle, emerging adult mosquitoes inherit common genetic traits from their parents with whom they share a very similar genetic profile. These individuals will later lay their eggs as adults in the same or surrounding breeding sites of the same locality with a high consanguinity. For this reason and in order to achieve a better appreciation of the genetic diversity of the populations, a few individuals from the breeding sites were randomly sampled in order to replicate, in the extent possible, the genetic structure that better reflects that of the underlying population from which the collected mosquitoes originate from.

The black dots in Fig. 1 indicate the different mosquito breeding sited prospected in each municipality. The samples collected at each site were grouped to form the population of each commune. Adult mosquitoes emerging from the reared larvae were stored in plastic tubes (Eppendorf 1.5 ml) at − 20 °C for subsequent molecular analysis.

### Molecular characterization of the collected mosquito samples

#### DNA extraction

Whole mosquitoes were crushed in 200 μl of 2% CTAB (20 g of cetyl trimethyl ammonium bromide; 100 ml Tris (Tris hydro methyl aminomethane) HCl 1 M; 20 ml of EDTA (Ethylene acid tetraacetic diamine) 0.5 M and 81.8 g of NaCl completed at 1 l with bi-distilled water). After 5 min in a water bath at 65 °C, the mash was then mixed with 200 μl of chloroform, and centrifuged at 12,000 revolutions per minute for 5 min. The resulting supernatant was delicately recovered in another tube and supplemented by 200 μl of isopropanol, thoroughly mixed by inversion then again, centrifuged at 12,000 revolutions per minute for 15 min. The liquid contained in the tube was carefully disposed of to preserve the pellet at the bottom of the tube. Next, 200 μl of 70% ethanol were added to this pellet for precipitation. After 5 min at 12,000 revolutions of centrifugation, the supernatant of the tube was again finely reversed. The pellet was then drained for at least 3 h on the bench. The extracted DNA was reconstituted with 20 μl of sterile water and left in suspension on the bench all night long.

#### Identification of the different species of *Anopheles gambiae* collected

The strong morphological similarity between the species of the *An. gambiae* complex is the source of confusion during the identification of the constituent taxa. To overcome this difficulty, it is necessary to supplement morphological studies with molecular analysis [36]. The PCR diagnostic approach was reportedly used to identify mosquito species in multiple studies. It is based on the characteristic and irreversible insertion of a transposable element of 230 pb (SINE200) on the X chromosome of *An. coluzzii* (form M), while it is absent in its twin *An. gambiae* (form S). This genetic hereditary feature allows unambiguous, simple and direct recognition of the molecular forms still called M and S [36].

**Fig. 2:**
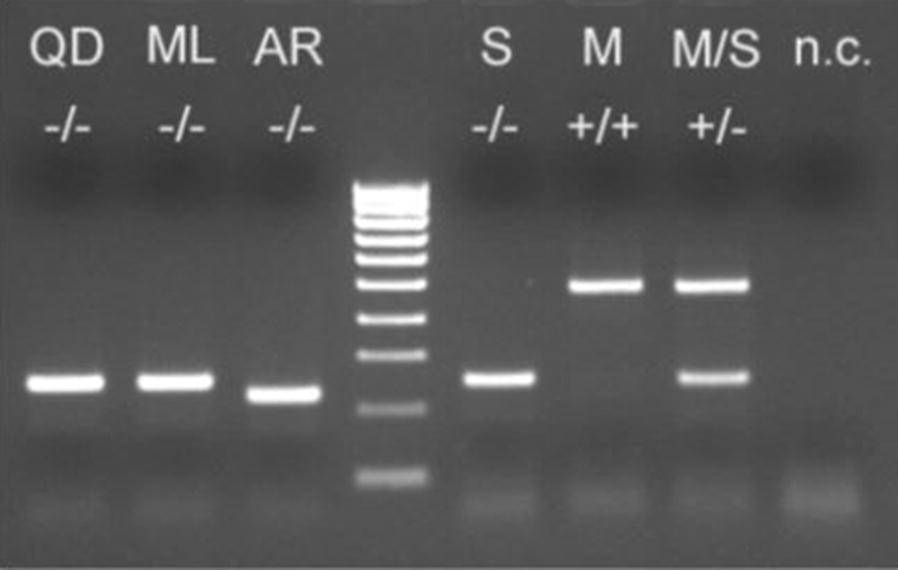
PCR diagnosis based on *S200* X6.1 in *Anopheles gambiae s.l*. PCR results from locus *S200* X6.1 indicating the presence (+) or absence (−) of the insertion in females of *Anopheles gambiae* species complex. QD = *A. Quadriannulatus* A; ML = *A. Melas*; AR = *A. arabiensis*; S = *A. gambiae* S-form; M = *A. gambiae* M-form; M/S = M/S hybrids from laboratory crosses; n.c. = negative control. Ladder = 100 bp (BIOLINE Hyper Ladder IV) [36]

The PCR protocol used includes an initial denaturation of the DNA material at 94 °C for 5 min followed by 35 cycles. Each cycle includes a denaturation phase at 94 °C for 30 s, a primer hybridization at 54 °C for 30 s, and an elongation phase through sequential nucleotide additions at 72 °C for 30 s. At the end of all cycles, a final elongation phase at 72 °C for 10 min is conducted to allow a total amplification of the sequences in progress. In addition to the conventional components of a chain polymerization reaction, the primers 200X6.1F TCG CCT TAG ACC TTG CGT TA and 200X6.1R CGC TTC AAG AAT TCG AGA TAC were used [36]. The amplification product obtained are stored at a final temperature of 4 °C before being migrated on a 1.5% agarose gel in the presence of ethidium bromide used as an intercalant and migration front. Figure 2, developed by Santolamazza et al. [36], illustrates the characteristic electrophoretic profile of each species.

The protocol of Santolamazza et al. [36] does not allow to differentiate *Anopheles quadriannulatus*, *Anopheles melas* and *An. gambiae* species because their electrophoretic profile shows bands that are similar at the same level, thus with almost the same molecular weight and by extension the same size. Since samplings were not done from lagoon coastal environments, it is logical to exclude from the analyzed samples, the existence of *An. melas*, a brackish-infeoded species. All mosquitoes were processed for molecular species identification using the Santolamazza et al. PCR protocol. All pre-identified mosquitoes were processed to a second PCR using the protocol of Scott et al. [37] to confirm the absence of other species from *An. gambiae s.l.*. The protocol consists in determining the polymorphisms in the intergenic spacer (IGS) of ribosomal DNA. It allows the identification of the different sibling species of the *An. gambiae* complex, namely *An. gambiae s.s.*, *Anopheles arabiensis*, *An. melas*, *Anopheles merus*, *An. quadriannulatus*, and *Anopheles bwambae*. For that, the following primers were used:

- UN: GTGTGCCGCTTCCTCGATGT
- AG: CTGGTTTGGTCGGCACGTTT
- AA: AAGTGTCCTTCTCCATCCTA
- ME: TGACCAACCCACTCCCTTGA
- QD: CAGACCAAGATGGTTAGTAT

The UN primer anneals to the same position of the rDNA of all five species, and AG anneals specifically to *An. gambiae*. ME anneals to both *An. merus* and *An. melas*. AA anneals to *An. arabiensis* and QD anneals to *An. quadriannulatus*. The PCR was carried out using a program of 30 cycles of denaturation at 94 °C for 30 s, annealing at 50 °C for 30 s, and extension at 72 °C for 30 s. Amplified DNA copies are stored at a final temperature of 4 °C prior to migration on a 2.5% agarose gel with ethidium bromide used as intercalating agent and migration front.

#### Detection of the *L1014F* mutation of the *kdr* gene

The presence of resistance alleles *(L1014F)* of the *kdr* gene in samples collected at each study site was tested using PCR, whose protocol and amplification program are described by Martinez-Torres et al. [21]. In fact, the PCR-PASA consists in using the specific primers named Agd1, Agd2, Agd3, Agd4 and Taq polymerase to search by special amplification for resistant or sensitive alleles on a fragment coding for *Vgsc* in each mosquito tested. The primer pair Agd1/Agd2 matches the *kdr* gene by amplifying a 293 bp product as a control. The primer pair Agd3/Agd1 is only matched to the resistance allele of the *kdr* gene to amplify a 195 bp fragment. Finally, the pair of Agd4/Agd2 primers associates only to the sensitive allele of the same gene by amplifying a 137 bp fragment. The nucleotide sequences of these primers are [21]:

- Agd1: 5′-ATAGATTCCCCGACCATG-3′;
- Agd2: 5′-AGACAGGATGAACC-3′;
- Agd3: 5′-AATTTGCATTACTTACGACA-3′;
- Agd4: 5′-CTGTAGTGATAGGAATTTA-3′.

#### Analysis of population genetic structure

Several analytical approaches were used to establish the genetic structure of populations. The genetic constitution of each population is determined by calculating the allelic and genotypic frequencies from the *kdr* gene. For this purpose, Genepop version 4.2 was used to calculate these frequencies. Via R version 3.3.3, The p-value associated with each gene frequency were calculated using the binomial law. The Hardy–Weinberg Equilibrium Test (EHW) was determined using Genetics software version 1.3.8.1 that allowed to check whether panmixia exists in the different populations of mosquitoes of the agroecological zones under study. Other indices allowing to identify the probable causes of a possible deviation from panmixia were calculated using the formulae of Weir and Cockerham [38] and Robertson and Hill [39], implemented in the Genepop version 4.2 software. These are observed heterozygoty (Ho), expected heterozygoty (He), expected numbers, fixation index (*F*_*IS*_) and genetic differentiation within populations (*F*_*ST*_) and between populations (*F*_*SC*_). Fixation index (*F*_*IS*_) is used to quantify the deviation to the panmixia. If *F*_*IS*_< 0, the population has an excess of heterozygous, otherwise (*F*_*IS*_> 0), it has a heterozygous deficit and for *F*_*IS*_ = 0, the observed heterozygosis is statistically consistent with that expected and the population is likely panmictic. The criteria used to assess genetic differentiation in populations are those defined by Hartl [40]. Depending on the values obtained, genetic differentiation is low if the F_ST_ is less than or equal to 0.05; moderated when its value is between [0.05; 0.15] and large when it is between [0.15; 0.25]. This differentiation is very large if its value is greater than 0.25. The multiple comparison Chi square test for proportions was performed with R version 3.3.3.

## Results

### Insecticide use survey

**Fig. 3:**
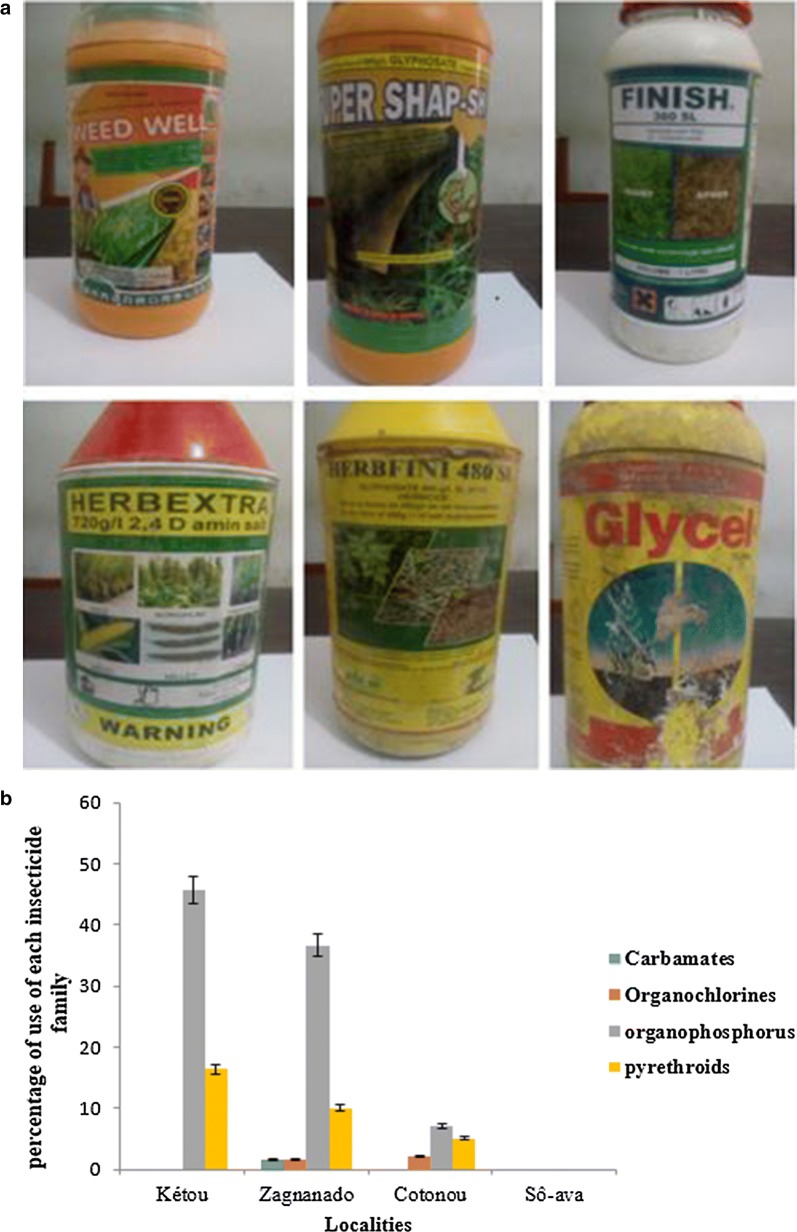
**a** Pictures of pesticide boxes identified during the surveys**. b** Percentage of various pesticide classed surveyed in each locality

Sixty farmers and household managers were asked about the pesticides they use. Sociological surveys have revealed that several classes of pesticides have been used in Zagnanado, Kétou and Cotonou. Those products have various commercial names, including Glyphosate 480, Herbfini, Herbex tra, Glycel, Finish, Force Up, Atrazine, Paraphosis, Fariapermefos EC, Lambda super, Pentagon, Para plus. Figure 3a displays pictures of some of the products used.

Pesticides identified during the surveys are of the classes of pyrethroids (Lambda super, Pentagon, Para plus, Para force), organophosphorus compounds (Killer 480 sl, Gramoquat super, Calriz, Adwuma Wura), organochlorines (endosulfan) and carbamates (Furadan). The proportions of each family of pesticides surveyed in the localities under study are shown in Fig. 3b.

As it can be seen in Fig. 3b, no insecticide class was reportedly used in Sô-Ava. However, this city has on several occasions, benefited from LLINs distribution campaigns. However, the effects of the various classes of pesticides, mainly pyrethroids, cannot be definitively excluded given their extensive use in public health for indoor residual spraying, and for the impregnation of distributed bed nets. With the exception of Sô-Ava, these classes of pesticides are not extensively used, organophosphates and pyrethroids are widely used in the other localities under study. Organochlorines are reported to be widely used in Cotonou and Zagnanado, while carbamates are only reported to be used in Zagnanado.

### Molecular characterization of the collected mosquitoes samples

The various species of the *An. gambiae* complex were sampled and the results are presented in Table 2. The results presented in Table 2 showed that the collected mosquitoes were *An. coluzzii, An. gambiae* or rarely their hybrids. Regardless of the locality, *An. coluzzii* was the majority species and *An. gambiae* was poorly represented. The *An. gambiae/An.coluzzii* hybrids were found only in Cotonou in statistically insignificant proportion. The relatively small number of *An. gambiae* obtained across the localities did not allow a robust statistical comparison of the proportions by the localities. For this reason, the study of the genetic structure was carried out only with *An. coluzzii.*

**Table 2:** Molecular identification of *An. gambiae* complex species

Localities	Collection sites (villages)	Sampling period	Latitude	Longitude	Number tested	*An.*	*An.*	*Hybrids*
						*coluzzii*	*gambiae*	*forms*
Cotonou	Fidjrossè	Rainy season	6°21′36.90″ N	2°22′15.08″ E	9	9	0	0
	Agla	Rainy season	6°23′01.36″ N	2°21′50.72″ E	8	8	0	0
	Vossa	Rainy season	6°23′21.57″ N	2°24′21.57″ E	20	17	1	2
	Menontin	Rainy season	6°23′34.85″ N	2°21′55.16″ E	10	10	0	0
	Kouhounou	Rainy season	6°23′20.26″ N	2°22′44.02″ E	5	5	0	0
	Vedoko	Rainy season	6°22′43.97″ N	2°23′17.26″ E	5	5	0	0
	Fifadji	Rainy season	6°23′52.42″ N	2°23′59.65″ E	9	9	0	0
	Zogbo	Rainy season	6°23′34.40″ N	2°23′32.89″ E	5	5	0	0
	Ste Rita	Rainy season	6°23′00.58″ N	2°23′50.65″ E	5	5	0	0
	Ladji	Rainy season	6°23′24.31″ N	2°25′43.89″ E	32	32	0	0
	Houéyiho	Rainy season	6°22′17.26″ N	2°23′50.47″ E	18	18	0	0
	Dandji	Rainy season	6°21′42.63″ N	2°29′42.62″ E	10	9	1	0
	Agbato	Rainy season	6°23′40.72″ N	2°26′37.42″ E	7	7	0	0
	Agbodjedo	Rainy season	6°23′33.46″ N	2°26′57.46″ E	9	8	0	1
	Yénawa	Rainy season	6°23′23.94″ N	2°27′24.26″ E	12	12	0	0
	Sènandé	Rainy season	6°22′42.86″ N	2°27′35.34″ E	6	5	1	0
	Ayélawadjè	Rainy season	6°23′33.48″ N	2°26′48.54″ E	7	5	2	0
	Gankpodo	Rainy season	6°23′21.29″ N	2°27′35.27″ E	6	6	0	0
	Total				183	175	5	3
Sô-Ava	Houndomè1	Dry season	6°28′18″24 N	2°24′06.83″ E	9	9	0	0
	Houndomè2	Dry season	6°28′16.52″ N	2°24′02.74″ E	10	10	0	0
	Dogodo	Rainy season	6°28′05.22″ N	2°24′57.09″ E	15	15	0	0
	Sô-Tchanhoué	Rainy season	6°28′33.45″ N	2°24′50.90″ E	21	21	0	0
	Ganvié	Rainy season	6°28′01.28″ N	2°24′58.99″ E	30	30	0	0
	Total				85	85	0	0
Kétou	Kétou centre	Rainy season	7°21′37.44″ N	2°36′14.24″ E	8	8	0	0
	Odometa (Oloka)	Dry season	7°21′13.39″ N	2°36′48.34″ E	32	28	4	0
	Kpankou	Rainy season	7°21′58.34″ N	2°36′17.87″ E	22	19	3	0
	Mowodani	Rainy season	7°21′42.88″ N	2°35′59.21″ E	28	25	3	0
	Total				90	80	10	0
Zagnanado	Zagnanado-centre	Rainy season	7°12′44.53″ N	2°20′31.99″ E	24	20	4	0
	Kpédékpo	Rainy season	7°12′40.56″ N	2°20′26.71″ E	18	15	3	0
	Bamé	Rainy season	7°12′47.82″ N	2°20′19.32″ E	18	17	1	0
	Houègbo-centre	Rainy season	7°12′39.86″ N	2°20′27.40″ E	14	12	2	0
	Total				74	64	10	0
Total					432	404	25	3

### Genotyping of the *Anopheles coluzzii* species and genetic structure of the populations

Using the site *L1014* of the *kdr* gene, the genotypes of *An. coluzzii* in each locality were determined and the different results are presented in Table 3. Resistant homozygous individuals (*L1014F/L1014F*) were strongly represented across the localities with values ranging from 55.294 to 92.572%. The highest rate was obtained in Cotonou (92.572%) while the lowest was reported in Sô-Ava (52.941%). In both heterozygotes (*L1014F/L1014L*) and sensitive homozygotes (*L1014L/L1014L*), the tendency is reversed with the highest proportions reported in Sô-Ava and the lowest in Cotonou. In each locality, the resistant homozygotes are highly represented, followed by the heterozygotes while the wild type homozygotes are poorly represented. From the Table 3 there is a significant difference accross municipalities (Cotonou, Sô-Ava, Zagnanado and Kétou) in resistant homozygous individuals (*L1014F/L1014F*) in Cotonou, Sô-Ava, Zagnanado and Ketou (χ^2^ = 58.442, df = 3, p value = 1.26510^−12^). A detailed pairwise comparison showed a significant difference in Cotonou-Sô-Ava, Cotonou-Kétou, Sô-Ava- Ketou, Sô-Ava- Ketou, Sô-Ava- Zagnanado (p value < 0.05), but there is no significant difference between Cotonou-Zagnanado (p value = 0.1959) and Zagnanado-Kétou (p value = 0.7867). Similar results were observed with the heterozygous individuals (*L1014L/L1014F*) across all localities. However, no significant difference was observed across municipalities in susceptible homozygous individuals (*L1014L/L1014L*).

**Table 3:** Genotyping of *An. coluzzii* individuals, and assessment of the allelic and genotypic frequencies

Localities	Number of genotype and frequencies in %			F %	p-value	p-value
	*L1014F/L1014F*	*L1014L/L1014F*	*L1014L/L1014L*	*(L1014F)*	*F(L1014F)*	(EHW)
Cotonou	162 (92.572)a	10 (5.714)a	3 (1.714)a	95.428	< 0.001	0.0026
Sô-Ava	45 (52.941)b	33 (38.824)b	7 (8.235)a	72.353	< 0.001	0.7882
Zagnanado	54 (84.376)ac	5 (7.812)ac	5 (7.812)a	88.281	< 0.001	< 0.001
Kétou	65 (81.250) c	10 (12.500) c	5 (5.250)a	87.500	< 0.001	< 0.001

*L1014F/L1014F*: resistant homozygous; *L1014L/L1014F*: heterozygous; *L1014L/L1014L: sensitive homozygous; F(*L1014F): Frequency of resistance allele; p-value *F*(L1014F): threshold of significance of the frequency of the allele *L1014F*; p-value (EHW): p-value at the Hardy-Weinberg Equilibrium

Frequencies with the same letter in columns do not differ significantly from one locality to another

On the one hand, the equilibrium of Hardy–Weinberg (EHW) has not been observed in the *An. coluzzii* population of Cotonou, Kétou and Zagnanado as it can easily be seen in Table 3 (p-value < 0.001). On the other hand, this panmixia seems to be observed in the *An. coluzzii* population of Sô-Ava (p-value = 0.7882). In order to confirm the EHW in Sô-Ava, the contingency test of χ^2^ at ddl = 1 was performed to allow us sufficient robustness and confidence on the conclusions to be drawn. For this purpose, the theoretically expected number of each genotype have been calculated. These numbers are 44.3964, 34.2071, 6.3964 for genotypes *L1014F/L1014F*, *L1014F/L1014L, L1014L/L1014L*, respectively.

χ^2^ = [(45 − 44.3964)^2^/(44,3964) + (33 − 34.2071)^2^/(34.2071) + (7 − 6.3964)^2^/(6.3964)] = 0.1077. The probability for ddl = 1 associated with this value of χ^2^ is 0.7427 which is both contained in the domain of validity and very close to that (0.7882) displayed by the p-value.

### Deviation from panmixia and genetic differentiation

Table 4 presents the observed heterozygoties *(Ho*) and expected heterozygoties (*He*), fixation index (*F*_*IS*_), genetic differentiations (*F*_*ST*_), in *An. coluzzii* populations across the localities.

**Table 4:** Difference in Panmixia, genetic differentiation within *An. coluzzii* populations

Localities	*Ho*	*He*	*F*_*IS*_ (W&C)	*F*_*ST*_ (W&C)
Cotonou	0.057	0.087	0.348	0.056
Sô-Ava	0.388	0.402	− 0.035 $\approx$ 0	− 0.018
Kétou	0.125	0.220	0.434	− 0.0049
Zagnanado	0.078	0.208	0.627	− 0.0464

*Ho*: observed heterozygoty; *He*: expected heterozygoty; *F*_*IS*_: fixation index; *F*_*ST*_: differentiation index; W&C: Weir & Cockerham

The observed heterozygosity was lower than that expected in the populations. EHW has not been observed in most populations. The heterozygosity deficit was widely shared among the *An. coluzzii* populations except for Sô-Ava. No genetic differentiation was observed in the *An. coluzzii* populations of Zagnanado and Ketou (*F*_*ST*_< 0). Nevertheless, this differentiation was moderate in Cotonou.

## Discussion

The surveys conducted on the utilization of insecticide identified four classes of pesticides in the localities with variable use against mosquito vectors and other pests. Except for Sô-Ava, pyrethroids and organophosphates are the mostly used classes of pesticides. This is not surprising since previous studies have shown in Benin that these two classes of insecticides are predominantly used in agriculture against crop pests, and also in public health in the fight against malaria-carrying insects [41]. The study results indicate a very low percentage of organochlorine use in Zagnanado. This class of insecticide is not even registered in the other localities. The probable reasons justifying this observation is the prohibition of organochlorine use in Benin due to its wide spread contamination of the ecosystem including surface waters and fish populations. In addition, organochlorines easily accumulate in sediments [42]. However, it may well be assumed that a sufficiently large increase in the size of the respondents would have revealed a higher use of this class of pesticide in the study localities. In Sô-Ava for instance, no insecticide use was reported. The absence of agricultural practices is probably the basis for such an observation. Similarly, the relatively low number of respondents is another probable justification of the reported absence of pesticide use in Sô-Ava. With human dwellings becoming increasingly modern, and the use of fine build materials, supplemental data on the use of LLINs are necessary to better assess pesticide selection pressure of resistant mosquitoes in this locality.

*Anopheles coluzzii* is strongly represented across all study localities. Corbel et al. [42] reported that *An. coluzzii* is the predominant species (> 98%) in southern Benin. In Cotonou and Sô-Ava, the constant presence of water favours the availability of breeding sites and the rapid reproduction of mosquitoes. In this study, molecular identification of the collected mosquito samples clearly shows that the different species live in sympathy with a predominance of *An. coluzzii,* thus confirming the same observation reported by other studies [43–45]. Mosquito larvae were harvested from standing water puddles, abandoned canoes and even heavily polluted pits throughout the study. Kudom showed in Ghana that *An. coluzzii* develops in polluted waters and in permanent and semi-permanent breeding sites [46]. This observation leads us to believe in ecological adaptation of the species likely related to current climate change. In the recent past, Gimoneau et al. [47] have reported that permanent mosquito breeding sites are the main habitats for *An. coluzzii,* while temporary breeding sites are colonized by *An. gambiae*. In Kétou and Zagnanado, the predominance of *An. coluzzii* still reflects the high distribution of this species in southern Benin as reported in previous studies [42]. The dominance of this species in Zagnanado can also be explained by the presence of permanent breeding sites on the rice production site. After molecular characterization of previously morphologically identified individuals, the *An. gambiae* species was poorly represented while its twin sister species *An. coluzzii* was dominant in all localities. The few numbers of *An. gambiae* collected could only be related to the collection method used (larvae collection) due to difficult access to the majority of *An. gambiae* breeding sites. Adult collections generally show a more diverse species composition than those observed in larvae collections.

The relatively high frequency of *L1014F allele* of the *kdr* gene observed in Sô-Ava despite the reported absence of pesticide use remains to be carefully investigated. The proximity of Cotonou to Sô-Ava may explain the relatively high pesticide selection of resistant mosquitoes in Sô-Ava. In fact, resistant mosquitoes could migrate from Cotonou to colonize the surroundings of Sô-Ava. For example, Nkya et al. [48] showed that a high frequency of resistance alleles of the *kdr* gene is associated with an overproduction of cytochrome P450 gene products (*CYP9J4* and *CYP6P1*) and alpha esterases AGAP006227 in a region where insecticide pressure is low but very close to an agricultural area in Kilimanjaro, Tanzania where the use of these synthetic substances is intense. It appears that this pattern of joint metabolic actions is conducive to the survival of the mosquito, and is one that is shared by populations of mosquitoes of the same lineage. Further Biochemical investigations on the detoxification of toxic chemicals are essential in populations of *An. coluzzii*. In the case of Sô-Ava, the use of long-lasting insecticidal nets (LLINs) with other unknown factors potentially favour the selection of resistant individuals in this locality. Pesticides use in vector control is considered to be the main cause of resistance emergence in mosquito populations [45, 49].

In Cotonou, a high frequency of *L1014F* allele was observed. Yadouleton et al. [12] showed that the use of pesticides in the market areas of Houéyiho located in Cotonou could lead to a strong selection of resistant individuals. The water availability associated with the hydromorphic nature of the soil of this locality (Fishery Zone) could lead to a rapid spread of selected resistant individuals in the market area and other unidentified locations. This would largely justify the rapid expansion of resistant individuals throughout the city. Several studies have shown that the use of insecticides against crop pests and the presence of pollutants in urban and industrial areas can play a significant role in the selection of resistance in mosquitoes [50]. Other studies have also showed that the use of pollutants in urban areas can influence the detoxification mechanisms of mosquitoes leading to better tolerance of mosquitoes to insecticides [51–53].

A high frequency of *L1014F* allele was also observed in Ketou and Zagnanado. The main crops in these areas were found to be cotton and rice, respectively [31, 32]. Producers use several classes of insecticides to protect their crops from pests. The development of agriculture through the massive use of pesticides is concurrently associated with a high level of resistance of malaria vectors to insecticides in West Africa [54–56]. In fact, most pesticides used by farmers are also used in public health for mosquito bed net impregnation and indoor spraying [12]. This practice potentially increases the selection pressure of resistant individuals in mosquito populations. The emergence of pyrethroid resistance in *An. gambiae* has become a major concern for the success of malaria control in the last decade [57, 58]. This insecticide class remains the only one used for mosquito net impregnation, the main control strategy against malaria vectors [59, 60]. At the molecular level, action sites for several classes of insecticides are widely shared by many taxa whether or not they are phylogenetic. Future biochemical and molecular studies could focus more on these aspects which could contribute to the improvement of knowledge on resistance mechanisms developed by local taxa.

In the municipalities of Cotonou, Zagnanado and Kétou, a high selection of the *L1014F* allele was observed, which was observed by the high frequency of the *L1014F/L1014F* genotypes. Several studies have shown in Benin, in the sub-region and throughout Africa that its resistance has been associated with high pesticide use. For example, in Burkina Faso, the work of Dabire et al. [61] showed that the link between agriculture and pyrethroid resistance was confirmed by a significant correlation between deltamethrin resistance levels and agricultural intensification in all populations. The authors discuss the fact that agriculture and urban areas are likely to promote the emergence of insecticide resistance. In agricultural areas, the main factor is the massive use of pesticides and in urban areas, uncontrolled indoor spraying of insecticides can strongly promote *kdr* mutations. Similarly, resistance to DDT and pyrethroids from *An. gambiae s.s.* populations in urban and agro-industrial environments has been observed in southern Cameroon [62].

At this stage, the genetic structure suggests that directional selection seems to be predominant in the populations of *An. coluzzii* exposed to insecticides and, in some cases, an almost total loss of molecular polymorphism was observed, with a frequency of the resistant allele *L1014F* exceeding 95%. In light of the results of this study and in the current environment, the selective value of resistant homozygotes *L1014F/L1014F* is likely higher than that of heterozygotes *L1014F/L1014L* which is in turn, higher than that of sensitive homozygotes *L1014L/L1014L.* In the current context, studies on the ecological adaptation of disease vectors need to be deepened in order to provide further clarification on mosquito ethology.

The EHW was observed in Sô-Ava. Since this locality is a lake city with a permanent presence of water and a strong wind action, it is not excluded that these factors mitigate or inhibit the effect of insecticides in the *Anopheles* population. This situation can explain the relatively high p-values (Table 3), suggesting a high risk of second-order error due to the apparently small sample size. Alternatively, the molecular marker used may be ill-suited and this study may have to go to a higher level of resolution such as nucleotide sequencing where the different haplotypes could be easily detected.

The fixation index calculated in the localities of Cotonou, Kétou and Zagnanado shows that all populations display a gap in panmixia. Heterozygous deficiency is generally observed in populations of these communities. It has been shown in population genetics that the causes of such an equilibrium gap of Hardy–Weinberg can be the Wahlund effect, inbreeding, genetic drift, selection against heterozygotes or their combination. Based on the results of the sociological surveys, it is tempting to believe in a situation of strong selection ofthe *L1014F* allele even if other factors may be involved. This observation would result in the effect of pesticides on mosquitoes which eliminate susceptible individuals leaving first-generation resistant individuals inbreed because of their limited population size. The resulting offspring will transmit almost exclusively the resistance allele to their descendants and, therefore, ensure the expansion of the resistance genetic mutation within the populations. Several studies have documented on the African continent, the role of chemical use in the development and spread of resistance. Such studies carried out in Benin [63], Cameroon [62], Sudan [64] and other African countries, have associated the increasing resistance of mosquitoes to insecticides with the increased use of pesticides.

No genetic differentiation was observed in the populations of mosquitoes of Zagnanado and Kétou. With these two localities being geographically close, this finding may be related to an apparent homogeneity of biotic and abiotic factors that could create genetic differentiation between individuals, and to some extent to the resolution power of the type of marker used. Moderate genetic differentiation in the population of mosquitoes of Cotonou may be rooted in the extrinsic factors capable of exerting, among others, insecticide pressures on the individuals of this population.

Genotypic differentiation within the different populations of the localities studied shows that genotypes are distributed similarly to Sô-Ava despite the fact the use of any class of pesticides was registered in that community. In one way or another, this observation suggests that there are several other unidentified factors which are involved in the distribution of *An. coluzzii*. The use of LLINs could also contribute to the selection of resistant individuals [65].

## Conclusions

The survey on the use of insecticides showed that insecticide selection pressures differ across the investigated localities. Although vector control programmes depend exceptionally on the use of residual insecticides, they should be preceded by a study of the genetic structure of vector populations because it depends essentially on the ecology of the environment and the genetic pool of vector populations. It would be desirable to rotate or apply formulations of combined products with different modes of action. Doing so would enable a better management of resistant homozygous individuals, and mitigate the resistance effect of commonly used insecticides. Further investigations using other methodological approaches such as microsatellites and nucleotide sequencing of appropriate genes providing a higher resolution, are necessary. Such deeper investigations may provide a better evaluation of the genetic differentiation within and between populations of disease vectors and assess the impact of the environment on their genetic structure and diversity.

## Data Availability

The data supporting the conclusions of this article are included within the article. The raw data used and/or analyzed in this study are available from the corresponding author upon reasonable request

## Abbreviations

WHO: World Health Organization
IRS: indoor residual spraying
*An.*: *Anopheles*
LLIN: long-lasting insecticidal nets
IRS: indoor residual spraying
DNA: desoxyribonucleic acid
CTAB: cetyl trimethyl ammonium bromide
PCR: polymerase chain reaction
*Kdr*: knock down resistance
*vgsc*: voltage gate sodium chanel
HWE: Hardy–Weinberg Equilibrium
IGS: intergenic spacer
W&C: Weir & Cockerham
*Ho*: observed heterozygosity
*He*: expected heterozygosity
*Fis*: fixation index
*Fst*: genetic differentiations within populations
*Fsc*: genetic differentiations between populations
